# How good are we at reporting the socioeconomic position, ethnicity, race, religion and main language of research participants? A review of the quality of reporting in palliative care intervention studies

**DOI:** 10.1177/02692163231224154

**Published:** 2024-02-08

**Authors:** Keerthika Selvakumaran, Katherine E Sleeman, Joanna M Davies

**Affiliations:** 1 GKT School of Medicine, King’s College London, London, UK; 2 Department of Palliative Care, Policy and Rehabilitation, Faculty of Nursing, Midwifery and Palliative Care, Cicely Saunders Institute, King’s College London, London, UK

**Keywords:** Quality of reporting social characteristics, intervention studies, socioeconomic position, race, ethnicity, religion, language, inequality in healthcare and society

## Introduction

There is social inequality in palliative and end-of-life care; people with lower socioeconomic position, from ethnic minorities and from other marginalised groups tend to experience worse care and outcomes towards the end of life, including having higher use of hospital-based care and less access to specialist palliative care.^1,2^ This pattern is observed across high-income countries and mirrors wider inequalities in health and society.^1^

Efforts to make palliative and end-of-life care more equitable are gaining traction.^2,3^ An important task within this equity-driven agenda should be to ensure that palliative care interventions are tested for safety and efficacy across different social groups.^4^ A lack of diversity in study participants limits the generalisability of evidence and can lead to the implementation of interventions that are ineffective for some groups or that exacerbate existing inequalities.^5–7^ Good quality reporting is essential for evaluating the diversity of participants.

In 2018, a review of 18 clinical trials on the integration of palliative care into oncology, found that one-third did not report the race or ethnicity of participants, and a further one-third provided only broad categorisations such as ‘white’ versus ‘other’.^8^ The aim of this pragmatic review is to describe the quality of reporting in palliative and end-of-life care intervention studies, for social characteristics including socioeconomic position, ethnicity or race, religion and the main language of participants.

## Method

We searched for primary studies in systematic reviews included in a ‘review of reviews’ on the effectiveness and cost effectiveness of palliative care, published by Luta et al1.^9^ Primary studies were included if they were quantitative studies of interventions (with or without a control group) published within a 5 year period before 2021 (i.e. from 2016 onwards). We extracted information on: (i) whether the study reported the age, sex, socioeconomic position, ethnicity or race, religion or main language of participants, (ii) the ethnic or racial diversity of the sample and (iii) whether the intervention was evaluated for subgroup effects across social groups. Information is presented separately for randomised controlled trials (RCT) and non-RCT intervention studies. All data was double extracted by two authors (Selvakumaran and Davies) with disagreement resolved through discussion.

## Results

We identified 25 eligible papers, 16 RCTs,^10–25^ and 9 non-RCT intervention studies (see supplemental material for full details).^26–34^ Sixteen studies were from the US, five from the UK, two from China and two from Australia (Table 1).

**Table 1.** The reporting of social characteristics in 25 intervention studies.

	All studies (*n* = 25)	RCTs (*n* = 16)	Non-RCTs (*n* = 9)
Socioeconomic position (SEP)			
Reported any measure of SEP, *n* (%)	15 (60%)	12 (75%)	3 (33%)
Median (range) *n* of categories^a^	5 (3,10)	5 (3,10)	5 (3,5)
Ethnicity or race			
Reported ethnicity or race, *n* (%)	16 (64%)	12 (75%)	4 (44%)
Median (range) *n* of categories	3.5 (2,7)	3.5 (2,7)	3.5 (2,6)
Reported binary categories, *n* (%)	3 (12%)	2 (13%)	1 (11)
Religion			
Reported religion, *n* (%)	3 (12%)	3 (19%)	0 (0%)
Median (range) *n* of categories	4 (2,6)	4 (2,6)	–
Main language			
Reported main language, *n* (%)	0 (0%)	0 (0%)	0 (0%)
Median (range) *n* of categories	–	–	–

RCT: randomised control trial.

a Where multiple measures of SEP are reported, this refers to the measure with the largest number of categories.

Studies were all published between 2016 and 2018. All studies reported the age and sex of participants. Ethnicity or race was reported in 64% of studies (75% of RCTs); SEP was reported in 60% studies (75% of RCTs). Of the 16 studies reporting race or ethnicity, on average 32% of participants were non-white, (12%–92%). Just one study (an RCT^35^) investigated the interaction between the effects of the intervention and participants ethnicity, no other subgroup effects were reported.

## Discussion

This pragmatic review found that important participant characteristics including ethnicity, race, socioeconomic position, religion and main language are often not reported in palliative care studies. A larger proportion of RCTs reported socioeconomic position, ethnicity or race and religion, compared to the non-RCT studies, which may have been due to greater control over data collection or more stringent reporting requirements for RCTs. The majority of studies reporting race or ethnicity (13 of 16) used more than two categories. However, in line with other reviews,^8^ we found a tendency towards reporting a small number of categories of ethnicity or race and socioeconomic position. The use of broad socioeconomic, ethnic or racial categories, particularly binary categories such as white versus non-white, are rarely adequate for understanding inequalities and should be avoided.^3,36^

This work provides a useful snap-shot of the quality of reporting based on studies included in a single ‘review of reviews’, a new systematic search was beyond scope. We did not report on measures of health literacy or religion or faith which were used in some studies and are important social characteristics to consider in the diversity of participants.^37,38^

Poor quality reporting of social characteristics hampers efforts to improve equity in palliative and end of life care. The development of guidelines, similar to examples seen elsewhere,^36^ could help to promote better quality reporting, and encourage more diverse participant recruitment in intervention studies in palliative care. As a minimum, guidelines should include the need for studies to: (i) report the socioeconomic position, ethnicity or race, religion, and the main language of participants, (ii) explain how data was collected and whether information was self-reported or assigned, (iii) avoid binary or broad categories and (iv) explain the reasons for limitations where adequate data reporting was not possible.

## Supplemental Material

sj-pdf-1-pmj-10.1177_02692163231224154 – Supplemental material for How good are we at reporting the socioeconomic position, ethnicity, race, religion and main language of research participants? A review of the quality of reporting in palliative care intervention studies Supplemental material, sj-pdf-1-pmj-10.1177_02692163231224154 for How good are we at reporting the socioeconomic position, ethnicity, race, religion and main language of research participants? A review of the quality of reporting in palliative care intervention studies by Keerthika Selvakumaran, Katherine E Sleeman and Joanna M Davies in Palliative Medicine
