# Supplementary material for: How good are we at reporting the socioeconomic position, ethnicity, race, religion and main language of research participants? A review of the quality of reporting in palliative care intervention studies
Source: Palliat Med. 2024 Feb 8;38(3):396–9. doi: 10.1177/02692163231224154 (PMC10955797; doi:10.1177/02692163231224154)
Supplement: sj-pdf-1-pmj-10.1177_02692163231224154 – Supplemental material for How good are we at reporting the socioeconomic position, ethnicity, race, religion and main language of research participants? A review of the quality of reporting in palliative care intervention studies [file sj-pdf-1-pmj-10.1177_02692163231224154.pdf]

Author, Year of Publication	Systematic Review paper was extracted from	title	Type of trial	link	country	intervention	primary outcome	age	sex	SEP	ethnicity	religion	language	SEP - measures used	n of SEP categories (** on item in previous column indicates the meaure with the highest number of categ[illegible] where more than	n of ethnicity categories	n of religion categories	n of language categories	% in non-white ethnic minority category/ies	Subgroup effects reported
1. Sudore (2017)	Ankuda and Meier	Effect of th	RCT	https://pub	United States	Patients reviewed either PREPARE plus an easy-to-read AD or the AD alone (no clinician/system-level intervention)	Easy-to-use, patient-facing ACP tools, without clinician- and/or system-level interventions, can increase planning documentation 25% to 35%	yes	yes	yes	yes	yes	no	Education<br>Finacial Instability<br>Social Standing**<br>Health literacy	10	6	2	n/a	43	yes - ethnicity ("There were no significant interaction effects observed for ACP documentation or ACP engagement as a function of age, gender, race/ethnicity, US acculturation, health literacy, presence of a surrogate decision maker, health
2. Bakitas (2017)	Dalta et al	Engaging p	RCT	https://ww	United States	an EPC feasibility study (4/1/14–8/31/15) for patients with NYHA Class III/IV HF and their caregivers in academic medical centers in the northeast and southeast U.S. The EPC intervention comprised: 1) an in-person	Patients experienced moderate effect size improvements in QOL, symptoms, physical, and mental health; caregivers experienced moderate effect size improvements in QOL, depression, mental health, and burden.	yes	yes	yes	yes	yes	no	Education**<br>Health insurance<br>Employment status	6	3	4	n/a	18	no
3. Carson (2016)	Bibas et al	Effect of Pa	RCT	https://ww	United States	Minimum of 2 family meetings led by. PC specialists to analyse improvement of family anxiet and depression	The primary outcome was Hospital Anxiety and Depression Scale symptom score (HADS; score range, 0 [best] to 42 [worst]; minimal clinically important difference, 1.5) obtained during 3-month follow-up interviews with the surrogate decision makers	yes	yes	yes	yes	yes	no	Health insurance	5	4	6	n/a	30	no
4. Curtis (2016)	Bibas et al	Randomize	RCT	https://pub	United States	Communication with trained faciliatator	Communication facilitators may be associated with decreased family depressive symptoms at 6 months, but we found no significant difference at 3 months or in anxiety or PTSD	yes	yes	yes	yes	no	no	Education	6	6	n/a	n/a	16	no
5. O'Donnell (2018)	Dalta et al	Social Wor	RCT	https://pub	United States	a structured evaluation of prognostic understanding, end-of-life preferences, symptom burden, and quality of life with routine review by a palliative care physician; communication of this information to treating clinicians; and longitudinal	Among the 31 survivors at 6 months, there was no measured difference between groups in depression, anxiety, or quality-of-life scores.	yes	yes	no	yes	no	no	n/a	n/a	2	n/a	n/a	26	no
6. White (2018)	Bibas et al	A Randomi	RCT	https://pub	United States	Multicomponent, family-support intervention delivered by the interprofessional ICU team		yes	yes	no	no	no	n/a	n/a	n/a	n/a	n/a	n/a	n/a	no
7. Farquahar (2016)	MA in Ankuda	The clinical	RCT	https://ww	UK	Breathlessness intervention service - utilising evidence-based non-pharmacological and pharmacological interventions to support patients with advanced disease in managing their breathlessness	BIS had a statistically non-significant effect for patients with non-malignant conditions, and slightly increased service costs, but had a qualitatively positive impact consistent with findings for advanced cance	yes	yes	yes	no	no	no	Employment Status	5	n/a	n/a	n/a	n/a	no

Author, Year of Publication	Systematic Review paper was extracted from	title	Type of trial	link	country	intervention	primary outcome	age	sex	SEP	ethnicity	religion	language	SEP - measures used	n of SEP categories (** on item in previous column indicates the meaure with the highest number of catego[illegible] where more than [illegible]	n of ethnicity categories	n of religion categories	n of language categories	% in non-white ethnic minority category/ies	Subgroup effects reported
						breathlessness														
8. Hanson (2017)	Ankuda and Meier	Effect of th	RCT	https://ww	United States	A GOC video decision aid plus a structured discussion with nursing home health care providers; attention control with an informational video and usual care planning	Primary outcomes at 3 months were quality of communication (QOC, questionnaire scored 0–10 with higher ratings indicating better quality)	yes	yes	yes	yes	no	no	Education level	5	3	n/a	n/a	14	no
9. Rogers (2017)	Dalta et al	Palliative (	RCT	https://pub	United States	whether an interdisciplinary palliative care intervention in addition to evidence-based HF care improves certain outcomes.	An interdisciplinary palliative care intervention in advanced HF patients showed consistently greater benefits in quality of life, anxiety, depression, and spiritual well-being compared with UC alon	yes	yes	no	yes	no	no	n/a	n/a	4	n/a	n/a	43	no
10. Wong (2016)	Dalta et al	Effects of a	RCT	https://pub	China	Home-based transitional palliative care for patients with end-stage heart failure (ESHF) after hospital discharge	There is evidence of the effectiveness of a postdischarge transitional care palliative programme in reducing readmissions and improving symptom control among patients with ESHF.	yes	yes	yes	no	no	no	Education** Employment Percieved economic status Housing type	4	n/a	n/a	n/a	n/a	no
11. Grudzen (2016)	MA in Ankuda	Emergency	RCT	https://pub	United States	ED-initiated palliative care consultation for patients with advanced cancer vs usual care took place	Emergency department-initiated palliative care consultation in advanced cancer improves quality of life in patients with advanced cancer and does not seem to shorten survival	yes	yes	yes	yes	no	no	Education Income** Health insurance	3	7	n/a	n/a	68	no
12. Steel (2016)	MA in Ankuda	A Web-bas	RCT	https://ww	United States	Patients with advanced cancer and family caregivers were randomized to a web-based collaborative care intervention or enhanced usual care.	The integration of screening and symptom management into cancer care is recommended.	yes	yes	yes	yes	no	no	Income	3	5	n/a	n/a	12	no
13. Johnson (2018)	Dalta et al	The feasibility of a randomised controlled trial to	RCT	https://ww	UK	palliative care clinic conducted by consultant cardiologist with special interest and heart failure nurse consultan	No difference in outcome reported	yes	yes	yes	no	no	no	Area-based deprivatic	5	n/a	n/a	n/a	n/a	no
14. Hopp (2016)	Dalta et al	Results of a	RCT	https://pub	United States	a prospective randomized intervention trial in patients with advanced HF who were hospitalized for acute decompensation at 3 urban hospitals, comparing the effect of palliative care consultation (PCC) with that of usual care	: In this predominantly African-American cohort of hospitalized patients with advanced HF, PCC did not lead to a greater likelihood of comfort care election compared with usual care. More robust palliative interventions should be	yes	yes	no	yes	no	no	n/a	n/a	2	n/a	n/a	92	no
[illegible]	[illegible]	[illegible]	[illegible]	[illegible]	[illegible]	[illegible]	[illegible]	[illegible]	[illegible]	[illegible]	[illegible]	[illegible]	[illegible]	[illegible]	[illegible]	[illegible]	[illegible]	[illegible]	[illegible]	[illegible]

Author, Year of Publication	Systematic Review paper was extracted from	title	Type of trial	link	country	intervention	primary outcome	age	sex	SEP	ethnicity	religion	language	SEP - measures used	n of SEP categories (** on item in previous column indicates the meaure with the highest number of categ where more thar	n of ethnicity categories	n of religion categories	n of language categories	% in non-white ethnic minority category/ies	Subgroup effects reported
						with that of usual care	palliative interventions should be													
15. Torke (2016)	Bibas et al	The Family	RCT	https://ww	United States	Dedicated trained nurse acting as a Family Navigator	A fully integrated nurse empowered to facilitate decision making is a feasible intervention in the ICU setting. It is well-received by ICU families and staff. A larger randomized controlled trial is needed to demonstrate an impact on important outcomes, such as surrogate	yes	yes	yes	yes	no	no	Education<br>Household income**	5	3	n/a	n/a	25	no
16. Tsianakas (2017)	Bradley	CanWalk: a	RCT	https://ww	UK	group walking intervention	Patient-reported outcome measures (PROMs) assessing QoL, activity, fatigue, mood and self-efficacy were completed at baseline and 6, 12 and 24 weeks.	yes	yes	yes	yes	no	no	Employmemt status**<br>Education<br>Owner Occupier	5	3	n/a	n/a	17	no
17. Shao (2017)	Ankuda and Meier	National P	Cohort Stu	https://pub	China	Public policy to create a new hospice benefit	The national policy changes fostering hospice care significantly increased hospice utilization, decreased invasive end-of-life care, and reduced the medical costs of terminal cancer patients.	yes	yes	no	no	no	no	n/a	n/a	n/a	n/a	n/a	n/a	no
18. Ersek (2017)	Ankuda and Meier	Association	Cohort Stu	https://acsj	United States	Developing inpatient hospice units and training palliative specialists	Aggressive care within the last month of life is common among patients with NSCLC and is associated with lower family evaluations of end-of-life care. Specialized care provided within an HPC unit may mitigate the negative effects of aggressive care on these	yes	yes	no	yes	no	no	n/a	n/a	6	n/a	n/a	28	no
19. Reilly (2016)	Brighton	Patients' e	Cross secti	https://ww	UK	A survey on all patients who had attended and completed the 6-week breathlessness support service intervention by sending them a postal questionnaire to self-complete covering experience, composition, effectiveness of the BSS and about	Patients' satisfaction with the breathlessness support service was high, and identified as important to this was a combination of personalised care, nature of staff, education and empowerment, and use of specific interventions. These components would be important in any	yes	yes	no	no	no	no	n/a	n/a	n/a	n/a	n/a	n/a	no
20. Garden (2016)	Dixon (2018)	The Bromh	Cohort stu	https://ww	UK	ACP	Hospital admissions: decrease from 202 to 91 (55%) admissions over two years.<br>Place of death: 67/68 residents died in preferred place.<br>Carer satisfaction: 64/80 carers who participated in ACP were surveyed (80% response rate); 92% rated service 9/10.	yes	yes	no	no	no	no	n/a	n/a	n/a	n/a	n/a	n/a	no
21. Younes (2017)	Ankuda and Meier	The Impact	Cohort Stu	https://jour	Australia	a community-based palliative care service (PCS).	, PCS was associated with reduced acute care admissions, bed days, and costs over the last year of life	yes	yes	yes	no	no	no	Socioeconomic Status	5	n/a	n/a	n/a	n/a	no

Author, Year of Publication	Systematic Review paper was extracted from	title	Type of trial	link	country	intervention	primary outcome	age	sex	SEP	ethnicity	religion	language	SEP - measures used	n of SEP categories (** on item in previous column indicates the meaure with the highest number of categ where more than	n of ethnicity categories	n of religion categories	n of language categories	% in non-white ethnic minority category/ies	Subgroup effects reported
22. Sudat (2017)	Ankuda and Meier	Impact of I	Cohort Stu	https://pub	United States	Community based palliative care	Advanced Illness Management has a positive impact on inpatient utilization, cost of care, hospice enrollment, and site of death	yes	yes	no	yes	no	no	n/a	n/a	2	n/a	n/a	22	no
23. Spilsbury	Ankuda and Meier	Community	Cohort Stu	https://ww	Australia	Community-based palliative care	Community based specialist palliative care was associated with hospital cost reductions across multiple life limiting conditions	yes	yes	yes	no	no	no	Health insurance<br>Type of residence**	3	n/a	n/a	n/a	n/a	no
24. Wang (2017)	Ankuda and Meier	Associatior	Cohort stu	https://ww	United States	Continous home care	CHC hospices had significantly lower rates of hospice disenrollment and post-hospice hospitalization, suggesting CHC service available may enable higher quality of end-of-life care.	yes	yes	yes	yes	no	no	Education**<br>Median household income**	5	4	n/a	n/a	12	no
25. May (2018)	May et al (2018)	Does Moda	Cohort stu	https://ww	United States	the effect of palliative care consultations (PCCs) and care in a palliative care unit (PCU) on cost ofcare, in comparison with usual care (UC) only and in comparison with each other	Both PCU and PCC are associated with lower hospital costs than UC. PCU is associated with a greater cost-avoidance effect than PCC, except where both interventions are provided early in the hospitalization. Both timely provision of PC for appropriate patients and creating	yes	yes	no	yes	no	no	n/a	n/a	3	n/a	n/a	52	no
